# Extended-Spectrum β-lactamase-Producing *Enterobacteriaceae* Shedding in Farm Horses Versus Hospitalized Horses: Prevalence and Risk Factors

**DOI:** 10.3390/ani10020282

**Published:** 2020-02-11

**Authors:** Anat Shnaiderman-Torban, Shiri Navon-Venezia, Ziv Dor, Yossi Paitan, Haia Arielly, Wiessam Abu Ahmad, Gal Kelmer, Marcus Fulde, Amir Steinman

**Affiliations:** 1 Koret School of Veterinary Medicine (KSVM), The Robert H. Smith Faculty of Agriculture, Food and Environment, The Hebrew University of Jerusalem, Rehovot 7610001, Israel; ashnaiderman@gmail.com (A.S.-T.); gal.kelmer@mail.huji.ac.il (G.K.); 2 Department of Molecular Biology, Faculty of Natural Sciences, Ariel University, Ariel 40700, Israel; shirinv@ariel.ac.il (S.N.-V.); zivddor@gmail.com (Z.D.); 3 The Miriam and Sheldon Adelson School of Medicine, Ariel University, Ariel 40700, Israel; 4 Department of Clinical Microbiology and Immunology, Sackler Faculty of Medicine, Tel Aviv University, Tel Aviv 6997801, Israel; yossi.paitan@clalit.org.il; 5 Clinical Microbiology Lab, Meir Medical Center, Kfar Saba 4428164, Israel; Ariellyhaya@clalit.org.il; 6 Braun School of Public Health and Community Medicine, Hebrew University, Jerusalem 9112102, Israel; wiessam@gmail.com; 7 Institute of Microbiology and Epizootics, Department of Veterinary Medicine at the Freie Universität Berlin, Berlin 14163 Germany; Marcus.Fulde@fu-berlin.de

**Keywords:** equine, ESBL-E, antibiotic resistance, shedding, risk factors, farm, ESBL-E acquisition

## Abstract

**Simple summary:**

This prospective study investigated the prevalence, molecular characteristics and risk factors of extended-spectrum β-lactamase (ESBL)-producing *Enterobacteriaceae* (ESBL-E) shedding in three equine cohorts: (i) farm horses (13 farms, n = 192); (ii) on admission to a hospital (n = 168) and; (iii) horses hospitalized for ≥72 h re-sampled from cohort (ii) (n = 86). Bacteria were isolated from rectal swabs, identified, antibiotic susceptibility patterns were determined, and medical records and owners’ questionnaires were analyzed for risk factor analysis. ESBL shedding rates significantly increased during hospitalization (77.9%, n = 67/86), compared to farms (20.8%, n = 40/192), and horses on admission (19.6%, n = 33/168). High bacterial species diversity was identified, mainly in cohorts (ii) and (iii), with high resistance rates to commonly used antimicrobials. Risk factors for shedding in farms included horses’ breed (Arabian), sex (stallion), and antibiotic treatment. Older age was identified as a protective factor. We demonstrated a reservoir for antibiotic-resistant bacteria in an equine hospital and farms, with a significant ESBL-E acquisition. In light of our findings, in order to control ESBL spread, we recommend conducting active ESBL surveillance programs alongside antibiotic stewardship programs in equine facilities.

**Abstract:**

We aimed to investigate the prevalence, molecular characteristics and risk factors of extended-spectrum β-lactamase (ESBL)-producing *Enterobacteriaceae* (ESBL-E) shedding in horses. A prospective study included three cohorts: (i) farm horses (13 farms, n = 192); (ii) on hospital admission (n = 168) and; (iii) horses hospitalized for ≥72 h re-sampled from cohort (ii) (n = 86). Enriched rectal swabs were plated, ESBL-production was confirmed (Clinical and Laboratory Standards Institute (CLSI)) and genes were identified (polymerase chain reaction (PCR)). Identification and antibiotic susceptibility were determined (Vitek-2). Medical records and owners’ questionnaires were analyzed. Shedding rates increased from 19.6% (n = 33/168) on admission to 77.9% (n = 67/86) during hospitalization (*p* < 0.0001, odds ratio (OR) = 12.12). Shedding rate in farms was 20.8% (n = 40/192), significantly lower compared to hospitalized horses (*p* < 0.0001). The main ESBL-E species (n = 192 isolates) were *E. coli* (59.9%, 115/192), *Enterobacter* sp. (17.7%, 34/192) and *Klebsiella pneumoniae* (13.0%, 25/192). The main gene group was CTX-M-1 (56.8%). A significant increase in resistance rates to chloramphenicol, enrofloxacin, gentamicin, nitrofurantoin, and trimethoprim-sulpha was identified during hospitalization. Risk factors for shedding in farms included breed (Arabian, OR = 3.9), sex (stallion, OR = 3.4), and antibiotic treatment (OR = 9.8). Older age was identified as a protective factor (OR = 0.88). We demonstrated an ESBL-E reservoir in equine cohorts, with a significant ESBL-E acquisition, which increases the necessity to implement active surveillance and antibiotic stewardship programs.

## 1. Introduction

Extended-spectrum beta-lactamase (ESBL)-producing *Enterobacteriaceae* (ESBL-E) poses a clinical challenge to both human and veterinary clinicians. ESBLs confer resistance to penicillins, cephalosporins, and aztreonam and are often accompanied by fluoroquinolone resistance, which even further narrows antibiotic treatment options [1]. Moreover, many ESBL genes are encoded on large plasmids, which enables lateral transfer between different bacterial species, within the same host and between different hosts [2]. In human medicine, ESBL production is associated with increased morbidity, higher overall and infection-related mortality, increased hospital length of stay, delay of targeted appropriate treatment, and higher costs [3,4]. Risk factors for colonization and infection in humans include severe illness with prolonged hospital stays, the presence of invasive medical devices for a prolonged duration and antibiotic use [2].

Within the last decade, a growing burden of ESBL-E in companion animals is being observed, both as gut colonizing bacteria and as infecting pathogens, causing wounds, respiratory, urogenital, gastro-intestinal, umbilical infections, and bacteremia [5,6,7,8]. Horses were described as carriers, as well as infected by ESBL-E, in equine clinics and in farm settings [9,10]. Prevalence of ESBL-producing *E. coli* carriage in horses varies between 4–44% in different European countries [11,12,13], with a lower carriage prevalence in equine riding centers in comparison with equine clinics [10]. In equine community settings, being stabled in the same yard with a recently hospitalized horse was identified as a risk factor for ESBL-producing *E. coli* carriage [14]. Risk factor analysis in the level of the farm revealed that the odds of being an ESBL/AmpC-producing *E. coli* premises were higher among riding schools than breeding premises, if premises housed a horse that had been medically treated with antibiotics within the last three months, and also in premises where the staff consisted of more than five persons [13]. However, risk factors for shedding of different ESBL-E species within horses were not yet reported.

We aimed to investigate and compare ESBL-E shedding in different equine cohorts, including farm horses, horses on admission to an equine hospital and during hospitalization, as well as to determine risk factors for shedding. We hypothesized that shedding rates increase during hospitalization, that previous antibiotic treatment is a risk factor for shedding and that shedding on admission and during hospitalization is associated with clinical signs, prolonged hospitalization, and severe outcome. 

## 2. Materials and Methods 

### 2.1. Equine Study Cohorts, Study Design, and Sampling Methods

This prospective study was performed on 13 farms throughout Israel and in the Koret School of Veterinary Medicine—Veterinary Teaching Hospital (KSVM-VTH). The study was approved by the Internal Research Review Committee of the KSVM-VTH (Reference numbers: KSVM-VTH/15_2015, KSVM-VTH/23_2015). Rectal swabs were collected from the horses with owner consent. On admission, sampling was performed prior to any medical treatment in the hospital. When horses survived and were not discharged, a second sample was taken 72 h post-admission. Farm horses were located in different regions of Israel to roughly represent the population.

### 2.2. Demographic and Medical Data 

For farm horses (cohort (i)), owners’ questionnaires were reviewed for data regarding individual horses, including the originating farm, signalment (age, sex, and breed), duration of the horse’s accommodation in the farm, hospitalization and antibiotic treatments within the previous year. 

For hospitalized horses (cohort (ii)), medical records were reviewed for the following information: signalment (age, sex, and breed), geographic origin, previous admission to the hospital within the previous year (yes/no), clinical signs, duration of illness before admission, antibiotic therapy before and during hospitalization, surgical procedures, other medications, hospitalization length, short-term outcome, and admission charge. 

### 2.3. ESBL-E Isolation and Species Identification

Rectal specimens [14] were collected using bacteriological swabs (Meus s.r.l., Piove di Sacco, Italy) and were inoculated directly into a Luria Bertani infusion enrichment broth (Hy-Labs, Rehovot, Israel) to increase the sensitivity of ESBL-E detection [15]. After incubation at 37 °C (18–24 h), enriched samples were plated onto Chromagar ESBL plates (Hy-Labs, Rehovot, Israel), at 37 °C for 24 h. Colonies that appeared after overnight incubation at 37 °C were recorded, and one colony of each distinct color was re-streaked onto a fresh Chromagar ESBL plate to obtain a pure culture. Pure isolates were stored at −80 °C for further analysis. 

Isolates were subjected to Vitek-MS (BioMérieux, Inc., Marcy-l’Etoile, France) for species identification or to Vitek-2 (BioMérieux, Inc., Marcy-l’Etoile, France) for species identification and/or antibiotic susceptibility testing (AST-N270 Vitek 2 card). Chloramphenicol, enrofloxacin, and imipenem were analyzed using disc diffusion assay (Oxoid, Basingstoke, UK). ESBL-production was confirmed by combination disk diffusion using cefotaxime and ceftazidime discs (Oxoid, Basingstoke, UK), as well as cefotaxime and ceftazidime with clavulanic acid (Sensi-Discs BD, Breda, The Netherlands). Results were interpreted according to the Clinical and Laboratory Standards Institute (CLSI) guidelines [16]. Multidrug-resistant (MDR) bacteria were defined as such due to their in vitro resistance to three or more classes of antimicrobial agents [17].

### 2.4. Molecular Characterization of ESBL-E

Isolates were examined for the presence of the *bla*CTX-M group using a multiplex polymerase chain reaction (PCR) from ESBL-E DNA lysates, as previously described [18]. Isolates that were found to be *bla*CTX-M PCR negative were further examined for the presence of *bla*OXA-1, *bla*OXA2, *bla*OXA10 [19], *bla*TEM, and *bla*SHV groups [20]. ESBL-producing *E. coli* isolates were subjected to PCR for the detection of *mdh* and *gyrB* genes in order to determine the presence of the worldwide pandemic *E. coli* ST131 lineage [21]. 

### 2.5. Sample Size and Statistical Analysis

The minimal sample size (number of animals sampled) for farm horses was calculated using WinPepi, based on an estimated shedding rate of 25% for ESBL-E in equine community livery premises [22] and on the fact that Israel is endemic for ESBL-E [23], with a confidence level of 95% and an acceptable difference of 7%, resulting in n = 147.

The minimal sample size for horses on admission to hospital was based on the expected difference between ESBL-E shedding and non-shedding horses and the percentage of admitted horses that were treated with antibiotics before admission since antibiotic treatment was assumed to be a risk factor for shedding [12]. Since there is no previous study revealing percentages of antibiotic-treated horses and ESBL shedding, data for this calculation was based on a human study [24]. Estimating that 25% of horses on admission are ESBL shedders (representing the equine community) and that 72% and 44% of horses were treated with antimicrobials within shedders and non-shedders, respectively, with a 5% significance level and power of 80%, the total required sample size is 145 horses, including 116 non-shedders and 29 shedders. 

Risk assessment was performed using Chi-square or Fisher’s exact tests for association between individual variables, shedding and ESBL-E acquisition. Descriptive statistics were used to describe shedding rates. Continuous variables were analyzed using t-tests or Mann–Whitney U-tests. *p* ≤ 0.05 was considered statistically significant. For risk factor analysis of farm horses, a logistic regression model (multivariable analysis) was conducted using all the significant variables in the univariable analysis at a significance level of *p* < 0.2 using the ENTER method (IBM SPSS Statistics 25). Categorical data were summarized by the number of cases (percentage) and confidence intervals (95%) were calculated by Fisher’s (WinPEPI 11.15 Describe A).

In order to compare between shedding rates and antibiotic resistance rates within horses on admission and during hospitalization (cohorts (ii) and (iii), respectively), a mixed effect logistic regression model was conducted (STATA version 13). Resistance was defined as complete resistance (not including “intermediate resistance”). Odds ratio (OR) for a significant change in antibiotic resistance rates is defined as OR for a change in one resistance category (e.g., a change from “susceptible” to “intermediate” or from “intermediate” to “resistant”). A comparison between shedding rates and antibiotic resistance rates between farm horses (cohort (i)) and horses on admission (cohort (ii)) was performed using Chi-square. 

## 3. Results

### 3.1. Characterization of the Equine Study Populations (Table 1)

Overall, 192 horses were sampled, originating from 13 farms across Israel (June 2016–September 2018). The average number of sampled horses per farm was 15 (range: 3–26 horses). 

On admission, 168 horses were sampled (November 2015 to April 2016). Horses were admitted to hospitalization due to the following reasons: gastro-intestinal pathologies (33%, n = 55/168), orthopedic disorders (17%, n = 29/168), healthy (mares of sick neonatal foals or foals of sick mares, 17%, n = 29/168), reproduction disorders (12%, n = 20/168), neonatology disorders (12%, n = 20/168), respiratory disorders (4%, n = 7/168), and others (including ophthalmic, hematology, endocrine, teeth disorders, and tumors, 5%, n = 8/168). The median length of illness before admission was one day (range: several hours–750 d). Horses hospitalized for ≥72 h were re-sampled (n = 86). 

### 3.2. Antibiotic Therapy, Surgical Procedures, Length of Stay, and Outcome

A proportion of 8.3% (n = 16/192) of farm horses was hospitalized within the previous year, ranging from 0–30% between farms. A proportion of 19.8% (n = 38/192) of horses were treated with antibiotics within the previous year, ranging from 0–61% between farms. On admission, 9.5% (n = 16/168) of horses were reported to be previously hospitalized (within a year period), and 16.1% (n = 27/168) of horses were treated with antibiotics within the previous year. Previous hospitalization and antibiotic treatment prevalence rates were not significantly different in comparison with farm horses. 

During hospitalization, 50.6% of horses (n = 85/168) were treated with antibiotics, a proportion which is significantly higher than antibiotic treatment in farms and prior to admission (*p* < 0.0001). Surgical procedures were performed in 36.9% of horses (n = 62/168). The median length of stay was three days (range: several hours-21 d). Out of all horses admitted to hospitalization, 84.4% survived to discharge (n = 142/168).

### 3.3. Prevalence of ESBL-E Shedding 

Within farm horses, shedding rate was 20.8% [n = 40/192, 95% Confidence interval (CI) 15.3–27.3%, Table 1]. Shedding rate on admission was 19.6% (n = 33/168, 95% CI: 13.9–26.5%), which was not statistically different from shedding rate in farms (*p* = 0.79). Shedding rate of hospitalized horses (re-sampled) was 77.9% (n = 67/86, 95% CI 67.7–86.1%), which was significantly higher than the shedding rate on admission and in farms (p<0.001, OR = 12.12, 95% CI 3.92–37.49). Out of 67 hospitalized shedding horses, 77.6% (n = 52/67, 95% CI 65.8–86.9%) did not shed ESBL-E on admission.

### 3.4. Distribution of ESBL-E Species and ESBL Genes

**Figure 1:**
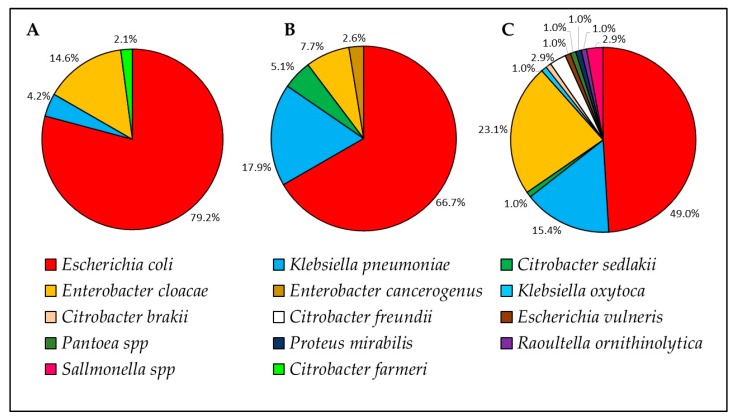
ESBL-E species distribution isolated from cohort (i) farm horses ((**A**), n = 48 isolates), cohort (ii) horses on admission to the hospital ((**B**), n = 39 isolates) and cohort (iii) 72 h post-admission ((**C**), n = 105 isolates).

Overall, 192 ESBL-E isolates were analyzed (Table A1). Fourteen bacterial species were identified of which three were identified in all cohorts—*E. coli*, *Klebsiella pneumoniae,* and *Enterobacter cloacae* (Figure 1). The most prevalent bacterial species in all cohorts was *E. coli*, consisting of 79.2% of isolates from farms, 66.7% from horses on admission, and 49.0% from hospitalized horses. However, the prevalence of *E. coli* decreased in horses on admission and in hospitalized horses, as the diversity of other ESBL-E species increased, from four species in farms to five species on admission and twelve species in hospitalized horses. Nosocomial ESBL-E species that were not identified in farms and on admission included *Citrobacter freundii* (n = 3/105), *Salmonella* spp (n = 3/105), *K. oxytoca*, *Citrobacter brakii*, *E. vulneris*, *Pantoea* spp, *Proteus mirabilis,* and *Raoultella ornithinolytica* (n = 1/105 each). The pandemic hypervirulent *E. coli* ST131 [25] was identified in three horses: two horses on admission and one horse during hospitalization. The main ESBL gene was the blaCTX-M-1 group in all cohorts (total 56.8% of all isolates, Table 2).

### 3.5. Antibiotic Susceptibility Profiles

Antibiotic resistance rates varied between cohorts, with a significant increase during hospitalization. All isolates from all cohorts were susceptible to imipenem (Table 3).

Among bacteria that grew on Chromagar ESBL plates, the prevalence of MDR bacteria was 89.6%, 71.8%, and 94.3% in farms, horses on admission, and hospitalized horses, respectively. The prevalence rate was significantly higher in isolates originated from hospitalized horses compared to horses on admission (*p* = 0.001, Table 2).

### 3.6. Risk Factor Analysis for ESBL-E Shedding

#### 3.6.1. Farm Horses

In univariable analysis, horses’ breed, sex, hospitalization in the previous year, antibiotic treatment in the previous year, and age were significantly associated with ESBL-E shedding (Table A2). Since the Arabian breed was the most prevalent breed sampled, we clustered all other breeds as one category in the multivariable analysis. In a logistic regression model, the breed (Arabian), sex (stallion versus mare, which was the reference in this category), and antibiotic treatment in the previous year were identified as risk factors for shedding. Age greater than one year was identified as a protective factor (Table 4).

#### 3.6.2. Horses on Admission

Signalment (age, sex, and breed), geographic origin, prior hospitalizations in the last year, clinical signs, length of illness before admission, antibiotic therapy before and during hospitalization, surgical procedures, other medications, hospitalization length, short-term outcome, and admission charge were not associated with ESBL-E shedding on admission (Table A2). Sex, hospitalization length, and admission charge resulted in *p* < 0.2, therefore, were analyzed via a logistic regression model, which did not yield any significant associations (Table A3).

#### 3.6.3. Horses During Hospitalization

There was no association between ESBL shedding 72 h post-admission and on admission, clinical signs on admission, antibiotic treatment during hospitalization, surgical procedures during hospitalization, length of stay, admission charge and outcome (Table A2). 

## 4. Discussion

This study investigates ESBL-E shedding in three equine cohorts, including farm horses, representing community equine, as well as horses on admission to the hospital and during hospitalization. Studies regarding antibiotic-resistant pathogens shedding, either in farm horses or in hospitalized horses were reported previously from different European countries [13,22,26,27]. Our study compares different equine cohorts within the same country. Both community and hospital cohorts are of great interest, from a veterinary and a ‘one health’ perspective, therefore it is highly valuable to compare these cohorts. 

We found high ESBL-E shedding rates (Table 2), an increased bacterial species diversity (Figure 1) as well as in the ESBL-E genes variety (Table 2). An increase in shedding rates may be due to the acquisition of bacteria, plasmids or resistance genes. The main bacterial species in all cohorts was *E. coli*, with decreased incidence on admission and during hospitalization, due to increased incidence of other nosocomial ESBL-producing bacterial species. The main ESBL gene group was CTX-M-1, as was previously reported in community horses [26]. However, on admission and during hospitalization, CTX-M-1 incidence decreases, alongside an increase in the number of ESBL genes. A study conducted in an equine hospital in the UK demonstrated the emergence of ESBL-producing *E. coli* during a decade [26], whereas we demonstrated a significant increase in ESBL-E shedding during individual horses’ hospitalization. These findings support an urgent necessity in active surveillance and infection control programs in veterinary facilities and hospitals. 

In addition, there is a need to set strict antibiotic stewardship programs in veterinary medicine, specifically in companion animals’ facilities, with specific guidance and enforcement. According to a recommendation published by the Committee for Medicinal Products for Veterinary Use (CVMP) of the European Union, there is a need to reserve fluoroquinolones, third and fourth generation cephalosporins for treatment when other options are likely to fail, and whenever possible, treatment should be supported by an antimicrobial susceptibility testing [28]. In practice, fluoroquinolones and cephalosporins are in use in equine medicine, sometimes as a first-line choice [29,30]. In our study, ESBL-E shedding as well as resistance rates for chloramphenicol, enrofloxacin, gentamicin, nitrofurantoin, and trimethoprim-sulpha increased significantly during hospitalization, resulting in a significant increase in MDR bacterial species shedding (Table 2 and Table 3). In light of our findings, as well as increasing resistance rates in other equine studies, we recommend implementing antibiotic stewardships in equine clinics and hospitals [31,32]. 

We also aimed to determine risk factors for shedding. We did not find significant associations between shedding on admission and during hospitalization to medical data. During the study period, we sampled all horses on admission, which represented a heterogeneous population, including critically ill horses alongside healthy mares, which were hospitalized together with their sick foals. Therefore, the lack of significant risk factors may be due to high variation in the equine population. Many of the pathologies on admission were attributed to the gastro-intestinal system, which might influence the intestinal microbiome. However, clinical signs on admission and during hospitalization were not associated with shedding. In farm horses, we detected several risk factors for ESBL-E shedding (Table 4). The Arabian breed was the main breed within farm horses and horses on admission to hospital. These horses in Israel are used mainly for breeding and shows and are held under intensive management, which may explain the risk for ESBL shedding. Interestingly, we detected the ‘stallion’ sex as a risk factor. In human medicine, it is reported that males are more susceptible to diverse bacterial illnesses than females, including an ESBL-E infection [33], presumably related to hormonal influences [34]. This may explain also our findings in veterinary medicine, however, it requires further investigation. Previous antibiotic treatment was identified as a risk factor as well, in agreement with other human and veterinary studies [2,13]. Age older than one year was identified as a protective factor, which may be due to the maturation of immunity. In a national survey of cattle farms in Israel, the prevalence of ESBL-E was higher in calves versus adult cows, where the use of antimicrobial prophylaxis was more common [35]. In human medicine, elderly age is associated with ESBL-E infections [33]. However, in our study, elderly horses older than 20 years old [36] were not prevalent and consisted of 3% (n = 12/360) of the study population. Therefore, elderly age may not be identified as a risk factor.

Our results should also be addressed from a ‘one health’ perspective. We detected resistant zoonotic bacteria both in farms and in hospital settings, which underlines the necessity for awareness and improved management. The human-animal interaction has great psychological and physical established benefits, with a great emphasis on equine-assisted therapy [37,38,39]. Therefore, there is pronounced importance in establishing safety policies involving therapists, physicians, and veterinarians, in order to ensure safe human-equine interactions in community settings [40]. This also applies to veterinary hospital staff. In a longitudinal study involving veterinary hospital staff and students, a higher level of ESBL-producing *E. coli* carriage was observed longitudinally [41], which underlines the necessity to implement gold standards biosecurity programs in veterinary hospitals.

## 5. Conclusions

Multi-drug resistant potentially zoonotic bacteria were detected both in farm horses and in hospitalized horses, with a significantly increased shedding during hospitalization. Therefore, we recommend implementing active surveillance programs alongside with infection control and antibiotic stewardship policies, in order to decrease resistance burden and to allow safe human-equine interactions.

## Figures and Tables

**Table 1:** Characterization of farm horses versus horses on admission to hospital.

Equine Cohort	Breeds ^1^	Median Age ^2^ (Years ± SD)	Sex Distribution ^3^
Farm horses (n = 192)	41.1% Arabians (n = 79/192) 25% pacers (n = 48/192) 15.1% Quarter horses (n = 29/192) 9.9% Warmbloods (n = 19/192) 5.2% local breed (n = 10/192) 3.7% ponies (n = 7/192)	8 ± 5.3	mares (72.4%, n = 139/192) geldings (12.5%, n = 24/192) stallions (11.5%, n = 22/192) ^4^
Horses on admission (n = 168)	49.4% Arabians (n = 83/168) 19.6% Quarter horses (n = 33/168) 14.3% pacers (n = 24/168) 7.7% Friesians (n = 13/168) 4.8% Warmbloods (n = 8/168) 4.2% others (n = 7/168)	4.5 ± 5.2	mares (68.5%, n = 115/168) geldings (16.1%, n = 27/168) stallions (15.4%, n = 26/168)

^1^ Breed distribution was not significantly different for Arabians, Quarter horses, and Warmbloods in comparison to farm horses, and was significantly different for the pacers horses (significantly higher in farms, *p* = 0.012) and Friesians (significantly higher on admission, *p* < 0.001); ^2^ Median age of horses on admission was significantly lower than the median age of farm horses (*p* < 0.0001); ^3^ Sex distribution was not significantly different between farm horses and horses on admission; ^4^ Data was not available for seven horses.

**Table 2:** Shedding rates of extended-spectrum beta-lactamase-producing *Enterobacteriaceae (*ESBL-E) in farm horses, on admission, and during hospitalization.

Equine Cohort	Shedding (%)	Total No. of ESBL-E Isolates	MDR Isolates (%)	*bla*ESBL Gene Group (%)
Farm horses	40/192 (20.8) (95% CI: 15.3–27.3%)	48	43/48 (89.6) (95% CI: 77.3–96.5)	CTX-M-1: 35/48 (72.9) CTX-M-9: 1/48 (2.1) CTX-M-25: 1/48 (2.1) SHV-12: 5/48 (10.4)
Horses on admission	33/168 (19.6) (95% CI: 13.9–26.5%)	39	28/39 (71.8) (95% CI: 55.1–85.0%)	CTX-M-1: 24/39 (61.5) CTX-M-9: 1/39 (2.5) SHV-12: 3/39 (7.7) SHV-2: 1/39 (2.5) SHV-28: 1/39 (2.5)
Hospitalized horses (72 h post admission) ^1^	67/86 (77.9) ^2^ (95% CI 67.7–86.1%)	105	99/105 (94.3) (95% CI: 87.9–97.9%) ^3^	CTX-M-1: 50/105 (47.6) CTX-M-2: 8/105 (7.6) CTX-M-9: 7/105 (6.7) CTX-M-25: 1/105 (0.95) OXA-1: 2/105 (1.9) SHV-12: 26/105 (24.7) SHV-228: 1/105 (0.95)

^1^ Horses re-sampled from cohort “horses on admission”; ^2^ Shedding rate in hospitalized horses is significantly higher than shedding rate on admission and in farms (*p* < 0.0001, OR=12.12, 95% CI 3.92–37.49); ^3^ Prevalence of multidrug-resistant (MDR) isolates is significantly higher in isolates originated from hospitalized horses compared to isolates originated from horses on admission (*p* < 0.001).

**Table 3:** Antibiotic ^1^ resistance rates (percentage) of ESBL-E isolates shed by farm horses, horses on admission, and hospitalized horses.

Equine Cohort	AMP	AMC ^2^	LEX	CAZ	IMP	CHL ^3^	ENR ^4^	AMK	GEN ^5^	NIT ^6^	TMS ^7^
Farms	100	41.7	100	100	0	66.6	6.3	0	75	4.2	89.6
On admission	100	82.1	100	85.0	0	46.2	17.9	2.6	48.7	5.3	76.3
During hospitalization	96.0	32.0	99.0	90.0	0	85.3	51.5	10.8	84.3	11.0	95.0

^1^ Abbreviations: ampicillin (AMP), amoxicillin-clavulanate (AMC), cephalexin (LEX), ceftazidime (CAZ), imipenem (IMP), chloramphenicol (CHL), enrofloxacin (ENR), amikacin (AMK), gentamicin (GEN), nitrofurantoin (NIT), and Trimethoprim- sulpha (TMS); ^2^ An increase in resistance rates for AMC on admission compared to farms (*p* = 0.001) and a decrease during hospitalization compared to admission (*p* < 0.001, OR = 0.1, 95% CI 0.04, 0.26); ^3^ An increase in resistance rates for CHL during hospitalization compared to admission (*p* < 0.001, OR = 6.5, 95% CI 2.8, 15); ^4^ An increase in resistance rates for ENR during hospitalization compared to admission (*p* < 0.001, OR = 4.2, 95% CI 1.9, 9.5); ^5^ An increase in resistance rates for GEN during hospitalization compared to admission (*p* < 0.001, OR = 12.3, 95% CI 2.9, 52.5); ^6^ An increase in resistance rates for NIT during hospitalization compared to admission (*p* < 0.001, OR = 3.7, 95% CI 1.4, 9.5); ^7^ An increase in resistance rates for TMS during hospitalization compared to admission (*p* < 0.01, OR = 6, 95% CI 1.9, 19.4).

**Table 4:** Risk factor analysis for ESBL-E shedding by farm horses (logistic regression model).

Variable	*p*-value	Odds Ratio (95% CI)
Breed (Arabian versus non-Arabian)	0.006	3.9 (1.5–10.4)
Sex (reference: mare)	0.079	-
Stallion	0.029	3.4 (1.1–12.2)
Gelding	0.744	0.7 (0.07–6.4)
Age	0.008	0.9 (0.8–0.97)
Hospitalization within the previous year	0.194	2.9 (0.6–14.8)
Antibiotic treatment within the previous year	<0.0001	9.8 (3.6–26.8)

## Appendix A

**Table A1:** Antimicrobial susceptibility profiles of individual isolates.

Num.	Horse Serial Number	Isolate	Origin	Bacterial ID	AMC	IMP	ENR	CHL	GEN	AMK	TMS	MDR
1	1	1.1.1	On admission	*Escherichia coli*	2	0	1	2	2	0	0	1
2	2	2.1.1		*Escherichia coli*	2	0	0	2	2	1	2	1
3	3	3.1.1		*Escherichia coli*	2	0	0	2	2	1	2	1
4	6	6.1.1		*Citrobacter sedlakii*	0	0	0	0	0	0	0	0
5	7	7.1.1		*Klebsiella pneumoniae*	2	0	1	2	2	0	2	1
6	15	15.1.1		*Escherichia coli*	2	0	1	2	2	1	2	1
7	15	15.1.2		*Klebsiella pneumoniae*	2	0	2	0	2	1	2	1
8	17	17.1.2		*Klebsiella pneumoniae*	2	0	1	0	0	0	2	1
9	22	22.1.1		*Escherichia coli*	2	0	0	2	2	1	2	1
10	22	22.1.2		*Klebsiella pneumoniae*	2	0	1	0	2	0	2	1
11	31	31.1.1		*Escherichia coli*	1	0	0	2	2	1	2	1
12	32	32.1.1		*Escherichia coli*	2	0	1	2	0	0	2	1
13	46	46.1.1		*Enterobacter cloacae*	2	0	0	0	0	0	0	0
14	60	60.1.1		*Escherichia coli*	2	0	1	0	0	0	2	1
15	74	74.1.2		*Enterobacter cloacae*	2	0	2	2	2	2	2	1
16	77	77.1.1		*Escherichia coli*	2	0	1	0	0	0	2	1
17	81	81.1.1		*Escherichia coli*	2	0	1	2	2	0	2	1
18	101	101.1.1		*Escherichia coli*	2	0	2	2	2	0	2	1
19	101	101.1.2		*Klebsiella pneumoniae*	2	0	1	0	0	0	2	0
20	107	107.1.1		*Escherichia coli*	2	0	1	0	0	0	2	1
21	112	112.1.1		*Enterobacter cloacae*	2	0	0	1	0	0	0	0
22	113	113.1.1		*Escherichia coli*	2	0	1	0	0	0	0	0
23	120	120.1.1		*Escherichia coli*	1	0	2	2	2	1	2	1
24	121	121.1.1		*Escherichia coli*	2	1	2	2	0	0	0	1
25	136	136.1.1		*Escherichia coli*	2	0	2	2	0	0	2	1
26	136	136.1.2		*Klebsiella pneumoniae*	2	0	2	0	2	1	2	1
27	144	144.1.1		*Escherichia coli*	2	0	1	2	2	1	2	1
28	153	153.1.1		*Escherichia coli*	2	0	1	0	0	0	2	0
29	162	162.1.1		*Escherichia coli*	2	0	0	0	0	1	0	1
30	176	176.1.1		*Escherichia coli*	2	0	1	2	2	1	2	1
31	177	177.1.1		*Escherichia coli*	1	0	1	2	2	0	2	1
32	179	179.1.1		*Escherichia coli*	1	0	0	2	2	0	2	1
33	203	203.1.1		*Klebsiella pneumoniae*	0	0	0	0	2	0	0	0
34	239	239.1.1		*Enterobacter cancerogenus*	2	0	0	0	0	0	0	0
35	244	244.1.1		*Citrobacter sedlakii*		0	0	0	0	0		0
36	267	267.1.1		*Escherichia coli*	2	0	1	0	0	0	2	1
37	278	278.1.1		*Escherichia coli*	2	0	1	0	0	0	2	1
38	288	288.1.1		*Escherichia coli*	2	0	1	0	0	0	2	1
39	290	290.1.1		*Escherichia coli*	2	0	1	0	0	0	2	1
40	1	1.2.2	During hospitalization	*Klebsiella pneumoniae*	0	0	1	0	2	0	2	1
41	5	5.2.1		*Escherichia coli*	0	0	0	2	2	0	2	1
42	6	6.2.1		*Klebsiella pneumoniae*	1	0	2	0	0	0	2	1
43	6	6.2.2		*Escherichia coli*	0	0	0	0	0	0	2	0
44	7	7.2.1		*Escherichia coli*	1	0	0	0	2	0	2	1
45	7	7.2.2		*Klebsiella pneumoniae*	1	0	2	2	2	0	2	1
46	8	8.2.1		*Escherichia coli*	2	0	1	2			2	1
47	8	8.2.2		*Enterobacter cloacae*	2	0	2	2	2	0	2	1
48	15	15.2.1		*Escherichia coli*	1	0	2	2	2	0	2	1
49	15	15.2.2		*Enterobacter cloacae*	2	0	1	2	2	0	2	1
50	16	16.2.1		*Escherichia coli*	0	0	1	2	0	0	2	1
51	16	16.2.2		*Enterobacter cloacae*	2	0	2	2	2	0	2	1
52	29	29.2.1		*Escherichia coli*	1	0	2	2	2	0	2	1
53	29	29.2.2		*Escherichia vulneris*	0	0	1	2	2	0	2	1
54	31	31.2.1		*Escherichia coli*	1	0	2	2	2	0	2	1
55	35	35.2.1		*Klebsiella pneumoniae*	1	0	1	2	2	0	2	1
56	46	46.2.1		*Pantoea spp*	1	0	2	2	2	0		1
57	46	46.2.2		*Escherichia coli*	1	0	2	2	2	0	2	1
58	47	47.2.1		*Escherichia coli*	1	0	2	2	2	0	2	1
59	47	47.2.2		*Enterobacter cloacae*	2	0	2	2	2	2	2	1
60	49	49.2.2		*Enterobacter cloacae*	2	0	1	2	2	2	2	1
61	55	55.2.1		*Escherichia coli*	1		1	2	2	0	2	1
62	55	55.2.2		*Klebsiella pneumoniae*	1	0	2	0	2	0	2	1
63	56	56.2.2		*Enterobacter cloacae*	2	0	2	2	2	1	2	1
64	57	57.2.1		*Escherichia coli*	0	0			0	0	2	0
65	60	60.2.1		*Enterobacter cloacae*	2	0	2	2	2	0	2	1
66	60	60.2.3		*Escherichia coli*	1	0	1	2	2	0	2	1
67	72	72.2.3		*Salmonella group*	1	0	0		2	2	2	1
68	75	75.2.3		*Enterobacter cloacae*	2	0	1	2	2	0	2	1
69	84	84.2.1		*Escherichia coli*	0	0	0	2	2	0	2	1
70	85	85.2.1		*Escherichia coli*	0	0	1	2	2	0	2	1
71	85	85.2.2		*Enterobacter cloacae*	2	0	2	2	2	0	2	1
72	87	87.2.1		*Escherichia coli*	0	0	2	2	0	0	2	1
73	87	87.2.2		*Escherichia coli*	1	0	2	2	2	0	2	1
74	89	89.2.1		*Escherichia coli*	1	0	2	2	2	0	2	1
75	89	89.2.2		*Klebsiella pneumoniae*	1	0	2	0	2	0	2	1
76	91	91.2.1		*Escherichia coli*	1	0	2	2	2	2	2	1
77	91	91.2.2		*Enterobacter cloacae*	2	0	2	2	2	0	2	1
78	101	101.2.1		*Escherichia coli*	1	0	2	2	1	0	2	1
79	101	101.2.2		*Klebsiella pneumoniae*	0	0	1	2	0	0	2	1
80	107	107.2.1		*Escherichia coli*	1	0	2	2	2	0	2	1
81	107	107.2.2		*Enterobacter cloacae*	2	0	2	2	2	2	2	1
82	107	107.2.4		*Enterobacter cloacae*	2	0	1	2	2	0	2	1
83	108	108.2.2		*Enterobacter cloacae*	2	0	1	2	2	0	2	1
84	113	113.2.1		*Escherichia coli*			2	2				
85	115	115.2.1		*Escherichia coli*	0	0	2	2	2	0	2	1
86	115	115.2.2		*Citrobacter freundii*	2	0	2	2	2	0	2	1
87	124	124.2.1		*Escherichia coli*	0	0	2	2	2	0	2	1
88	124	124.2.3		*Salmonella enterica*	1	0			2	2	2	1
89	126	126.2.2		*Citrobacter brakii*	2	0	2	2	2	1	2	1
90	127	127.2.1		*Escherichia coli*	1	0	2	2	2	1	2	1
91	127	127.2.2		*Enterobacter cloacae*	2	0	1	2	2	0	2	1
92	136	136.2.1		*Escherichia coli*	1	0	2	2	2	1	2	1
93	136	136.2.2		*Klebsiella pneumoniae*	1	0	2	0	2	0	2	1
94	143	143.2.1		*Escherichia coli*	0	0	0	2	2	0	2	1
95	143	143.2.2		*Citrobacter freundii*	2	0	0	2	2	0	2	1
96	144	144.2.1		*Escherichia coli*	2	0	1	2	2	0	2	1
97	144	144.2.2		*Citrobacter freundii*	2	0			2	1	2	1
98	144	144.2.3		*Proteus mirabilis*	1	0			2	0	0	1
99	148	148.2.1		*Escherichia coli*	2	0	0	0	0	0	2	1
100	149	149.2.1		*Escherichia coli*	0	0	0	2	2	0	2	1
101	149	149.2.2		*Enterobacter cloacae*	2	0	1	2	2	2	2	1
102	152	152.2.2		*Escherichia coli*	1	0	2	0	2	0	2	1
103	156	156.2.1		*Enterobacter cloacae*	2	0	1	2	2	2	2	1
104	156	156.2.2		*Escherichia coli*	1	0	2	2	2	0	2	1
105	158	158.2.2		*Klebsiella pneumoniae*	1	0	2	0	2	0	2	1
106	161	161.2.1		*Escherichia coli*	1	0	2	2	2	0	2	1
107	161	161.2.2		*Enterobacter cloacae*	2	0	1	2	2	0	2	1
108	167	167.2.1		*Klebsiella pneumoniae*	1	0	1	2	2	0	2	1
109	176	176.2.1		*Escherichia coli*	1	0	0	2	2	0	2	1
110	177	177.2.1		*Escherichia coli*	1	0	1	2	2	0	2	1
111	177	177.2.2		*Enterobacter cloacae*	2	0	1	2	2	0	2	1
112	181	181.2.1		*Enterobacter cloacae*	2	0	1		2	1	2	1
113	181	181.2.2		*Escherichia coli*	1	0	2	2	2	0	2	1
114	183	183.2.1		*Klebsiella pneumoniae*	1	0	1	2	2	0	2	1
115	183	183.2.2		*Escherichia coli*	1	0	0	2	2	0	2	1
116	195	195.2.1		*Escherichia coli*	1	0	1	2	2	0	2	1
117	212	212.2.1		*Escherichia coli*	0	0	1		2	0	0	0
118	216	216.2.1		*Escherichia coli*	0	0	2	2	0	0	2	0
119	219	219.2.1		*Escherichia coli*	1	0	2		2	0	2	1
120	222	222.2.1		*Klebsiella pneumoniae*	1	0	1	2	2	0	2	1
121	222	222.2.2		*Escherichia coli*	0	0	2	2	0	0	2	1
122	223	223.2.1		*Escherichia coli*	1	0	2	2	2	0	0	1
123	224	224.2.1		*Escherichia coli*	1	0	2	2	2	0	2	1
124	224	224.2.2		*Klebsiella pneumoniae*	1	0	2	2	2	0	2	1
125	228	228.2.1		*Klebsiella pneumoniae*	0	0	2	0	2	0	0	1
126	229	229.2.1		*Escherichia coli*	1	0	2	2	2	2	2	1
127	229	229.2.2		*Salmonella enterica*	1	0	2	2	2	2	2	1
128	234	234.2.1		*Raoultella ornithinolytica*	2	0	1	2	2	2	2	1
129	237	237.2.1		*Escherichia coli*	0	0	0	2	0	0	2	1
130	238	238.2.1		*Klebsiella pneumoniae*	1	0	1	0	2	0	2	1
131	243	243.2.1		*Escherichia coli*	0	0	2	2	0	0	2	1
132	243	243.2.2		*Enterobacter cloacae*	2	0	1	2	2	0	2	1
133	246	246.2.1		*Escherichia coli*	1	0			1	0	2	1
134	246	246.2.2		*Enterobacter cloacae*	2	0	2	2	2	0	2	1
135	265	265.2.1		*Enterobacter cloacae*	2	0	1	2	2	0	2	1
136	272	272.2.1		*Enterobacter cloacae*	2	0	1	2	2	0	2	1
137	273	273.2.1		*Enterobacter cloacae*	2	0	1	2	2	0	2	1
138	278	278.2.1		*Escherichia coli*	0	0	1	2	0	0	2	1
139	278	278.2.2		*Citrobacter sedlakii*	0	0	2	2	0	0		1
140	278	278.2.4		*Klebsiella pneumoniae*	0	0	2	2	2	0	0	1
141	279	279.2.1		*Klebsiella oxytoca*	2	0	0	0	2	1	2	1
142	279	279.2.2		*Escherichia coli*	0	0	1	2	0	0	2	1
143	289	289.2.1		*Escherichia coli*	1	0	2	0	2	0	2	1
144	H40	H40.2	Farms	*Escherichia coli*	2	0	1	0	0	0	2	1
145	H42	H42.1		*Escherichia coli*	2	0	1	2	2	1	2	1
146	H44	H44.1		*Escherichia coli*	2	0	1	2	2	1	2	1
147	H45	H45.2		*Citrobacter farmeri*	1	0	1	2	2	0	2	1
148	H48	H48.2		*Escherichia coli*	2	0	2	2	2	0	2	1
149	H48	H48.3		*Enterobacter cloacae*	2	0	1	2	2	1	2	1
150	H53	H53.1		*Escherichia coli*	2	0	1	0	0	0	2	1
151	H53	H53.2		*Enterobacter cloacae*	2	0	1	2	2	1	2	1
152	H54	H54.1		*Escherichia coli*	2	0	2	2	2	0	2	1
153	H56	H56.1		*Escherichia coli*	2	0	1	2	2	1	2	1
154	H56	H56.2		*Enterobacter cloacae*	2	0	1	2	2	1	2	1
155	H57	H57.1		*Enterobacter cloacae*	2	0	1	2	2	1	2	1
156	H57	H57.2		*Escherichia coli*	2	0	1	2	2	1	2	1
157	H60	H60.2		*Escherichia coli*	2	0	1	0	0	0	2	1
158	H110	H110.1		*Enterobacter cloacae*	2	0	0	0	0	0	0	0
159	H138	H138.1		*Escherichia coli*	2	0	0	0	0	0	0	1
160	H140	H140.1		*Escherichia coli*	2	0	0	2	2	1	2	1
161	H154	H154.2		*Klebsiella pneumoniae*	2	0	1	1	2	0	0	1
162	H157	H157.2		*Klebsiella pneumoniae*	2	0	2	0	0	0	2	1
163	H230	H230.1		*Escherichia coli*	1	0	1	2	2	0	2	1
164	H230	H230.2		*Enterobacter cloacae*	2	0	1	2	2	0	0	1
165	H231	H231.1		*Escherichia coli*	0	0	1	0	0	0	0	0
166	H233	H233.1		*Escherichia coli*	1	0	1	2	2	0	2	1
168	H234	H234.1		*Escherichia coli*	0	0	1	2	2	0	2	1
169	H234	H234.2		*Enterobacter cloacae*	2	0	1	2	2	0	2	1
170	H235	H235.1		*Escherichia coli*	1	0	1	2	2	0	2	1
171	H236	H236.1		*Escherichia coli*	0	0	1	2	2	0	2	1
172	H237	H237.1		*Escherichia coli*	1	0	1	2	2	0	2	1
173	H238	H238.1		*Escherichia coli*	0	0	1	2	2	0	2	1
174	H241	H241.1		*Escherichia coli*	1	0	1	2	2	0	2	1
175	H242	H242.1		*Escherichia coli*	1	0	1	2	2	0	2	1
176	H243	H243.1		*Escherichia coli*	1	0	1	2	2	0	2	1
177	H245	H245.1		*Escherichia coli*	1	0	1	2	2	0	2	1
178	H246	H246.1		*Escherichia coli*	1	0	1	2	2	0	2	1
179	H247	H247.1		*Escherichia coli*	0	0	1	2	2	0	2	1
180	H248	H248.1		*Escherichia coli*	0	0	1	2	2	0	2	1
181	H250	H250.1		*Escherichia coli*	0	0	1	2	2	0	2	1
182	H251	H251.1		*Escherichia coli*	0	0	1	2	2	0	2	1
183	H253	H253.1		*Escherichia coli*	0	0	1	2	2	0	2	1
184	H254	H254.1		*Escherichia coli*	0	0	1	2	2	0	2	1
185	H256	H256.1		*Escherichia coli*	0	0	1	0	0	0	2	1
186	H257	H257.1		*Escherichia coli*	1	0	0	1	2	0	2	1
187	H258	H258.1		*Escherichia coli*	0	0	0	0	0	0	2	0
188	H259	H259.1		*Escherichia coli*	0	0	0	0	0	0	2	0
189	H263	H263.1		*Escherichia coli*	0	0	0	0	0	0	2	0
190	H265	H265.1		*Escherichia coli*	1	0	0	1	2	0	2	1
191	H267	H267.1		*Escherichia coli*	0	0	1	0	0	0	2	1
192	H268	H268.1		*Escherichia coli*	1	0	0	1	2	0	2	1

Susceptible = 0, intermediate susceptibility = 1, resistant = 2. Empty cells mean lack of susceptibility test results due to technical reasons.

**Table A2:** Results of univariable analysis of variables gleaned from the medical records (horses on admission and during hospitalization) and owners’ questionnaires (farm horses). Variables were evaluated for association with the outcome of ESBL-E shedding status of the individual animal.

Population Studied	Variable	Classification	*p*-Value
Farm horses	Breed	Quarter Horse Arabian Pacer Warmblood Pony Local	<0.0001
	Sex	Female Male Gelding	0.027
	Farm	Numbered 1–13	
	Hospitalization within the previous year	Yes/No	0.018
	Antibiotic treatment within the previous year	Yes/No	<0.0001
	Age	Ranged from 0.1–23 y	<0.0001
	Time in farm	Ranged from 0–23 y	0.36
On admission	Breed	Quarter Horse Arabian Tennessee Walking horse Friesian Mangalarga Marchador Warmblood Thoroughbred Miniature horse Haflinger Hannoverian Single footed horse Missouri Fox Trotter	0.394
	Age	Years	0.259
	Sex	Female Male Gelding	0.117
	Geographical origin (within the country)	North South Center	0.879
	Hospitalization within the previous year	Yes/No	0.295
	Clinical signs on admission	Gastro-intestinal disorder Neonatology disorder Ophthalmic disorder Reproduction Orthopedic disorder Hematological disorder Respiratory disorder Endocrine disorder Healthy (mares of sick hospitalized foals)	0.587
	Length of illness before admission	Days	0.618
	Antibiotic treatment within the previous year	Yes/No	0.587
	Length of stay	Days	0.169
	Admission charge	-	0.056
During hospitalization	Shedding on admission	Yes/No	0.9
	Clinical signs on admission	Gastro-intestinal disorder Neonatology disorder Ophthalmic disorder Reproduction Orthopedic disorder Hematological disorder Respiratory disorder Endocrine disorder Tumor Teeth lesion Healthy (mares of sick hospitalized foals)	0.428
	Antibiotic treatment during hospitalization	Yes/No	0.841
	Outcome	Discharged/Died	0.174
	Length of stay	Days	0.29
	Admission charge	-	0.69

**Table A3:** Risk factor analysis for ESBL-E shedding by horses on admission to hospital (logistic regression).

Risk Factor	*p*-Value	OR
Sex (reference: mare)	0.647	
Stallion	0.409	0.571 (95% CI 0.151–2.162)
Gelding	0.639	0.765 (95% CI 0.25–2.34)
Length of stay	0.766	1 (95% CI 0.997–1)
Admission charge	0.184	1 (95% 1–1)
